# NGF-BMSC-SF/CS composites for repairing knee joint osteochondral defects in rabbits: evaluation of the repair effect and potential underlying mechanisms

**DOI:** 10.1186/s13018-024-04801-0

**Published:** 2024-07-29

**Authors:** Yong Zhang, Wenliang Huang, Hongli Xiao, Shiqiang Ruan, Jiang Deng

**Affiliations:** 1 The Third Affiliated Hospital of Zunyi Medical University (The First People’s Hospital of Zunyi), Zunyi City, Guizhou Province 563000 China; 2 The People’s Hospital of Bozhou District, Zunyi City, Guizhou Province 563000 China; 3 The First People’s Hospital of Guiyang City, Guiyang, Guizhou Province 550002 China

**Keywords:** Nerve growth factor, Bone marrow-derived mesenchymal stem cells, Silk fibroin, Chitosan scaffold, Tissue engineering, Cartilage damage, Osteoarthritis

## Abstract

**Background:**

With the rapid growth of the ageing population, chronic diseases such as osteoarthritis have become one of the major diseases affecting the quality of life of elderly people. The main pathological manifestation of osteoarthritis is articular cartilage damage. Alleviating and repairing damaged cartilage has always been a challenge. The application of cartilage tissue engineering methods has shown promise for articular cartilage repair. Many studies have used cartilage tissue engineering methods to repair damaged cartilage and obtained good results, but these methods still cannot be used clinically. Therefore, this study aimed to investigate the effect of incorporating nerve growth factor (NGF) into a silk fibroin (SF)/chitosan (CS) scaffold containing bone marrow-derived mesenchymal stem cells (BMSCs) on the repair of articular cartilage defects in the knees of rabbits and to explore the possible underlying mechanism involved.

**Materials and methods:**

Nerve growth factor-loaded sustained-release microspheres were prepared by a double emulsion solvent evaporation method. SF/CS scaffolds were prepared by vacuum drying and chemical crosslinking. BMSCs were isolated and cultured by density gradient centrifugation and adherent culture. NGF-SF/CS-BMSC composites were prepared and implanted into articular cartilage defects in the knees of rabbits. The repair of articular cartilage was assessed by gross observation, imaging and histological staining at different time points after surgery. The repair effect was evaluated by the International Cartilage Repair Society (ICRS) score and a modified Wakitani score. In vitro experiments were also performed to observe the effect of different concentrations of NGF on the proliferation and directional differentiation of BMSCs on the SF/CS scaffold.

**Results:**

In the repair of cartilage defects in rabbit knees, NGF-SF/CS-BMSCs resulted in higher ICRS scores and lower modified Wakitani scores. The in vitro results showed that there was no significant correlation between the proliferation of BMSCs and the addition of different concentrations of NGF. Additionally, there was no significant difference in the protein and mRNA expression of COL2a1 and ACAN between the groups after the addition of different concentrations of NGF.

**Conclusion:**

NGF-SF/CS-BMSCs improved the repair of articular cartilage defects in the knees of rabbits. This repair effect may be related to the early promotion of subchondral bone repair.

## Introduction

With the rapid growth of the ageing population, chronic diseases such as osteoarthritis have become one of the major diseases affecting the quality of life of elderly people [1]. The main pathological manifestation of osteoarthritis is articular cartilage damage. Alleviating and repairing damaged cartilage has always been a challenge [2]. The application of cartilage tissue engineering methods has shown promise for articular cartilage repair [3]. Articular cartilage has no direct blood supply and lacks nerves and lymphatics [4]. The joint cavity is a hypoxic environment, and its nutritional status mainly depends on the diffusion of surrounding synovial fluid and the blood supply to the subchondral bone; therefore, it is challenging for articular cartilage to repair itself once damaged [5]. There are multiple ways to treat cartilage damage [6–8], but clinically, no single treatment can completely treat this condition [9]. With the development of cross-disciplinary medicine in recent years, research and progress in the field of tissue engineering have shown promise for articular cartilage repair [10]. Tissue engineering is a discipline that uses engineering and life science technologies to repair or replace damaged human tissue and organs [11]. Several basic experimental studies have confirmed that cartilage tissue engineering has a certain effect on the repair of articular cartilage [12]. In our previous experimental study, we found that a silk fibroin (SF)/chitosan (CS) scaffold combined with bone mesenchymal stromal/stem cells (BMSCs) promoted the repair of articular cartilage defects in rabbits [13]. To further promote articular cartilage repair, we wanted to determine whether adding cytokines to the SF/CS scaffold-BMSC composite can increase the repair effects and achieve better therapeutic results.

The repair of articular cartilage damage can be promoted by the addition of cytokines for tissue engineering [14], such as transforming growth factors (TGFs) [15], insulin-like growth factor (IGF ) [16], and bone morphogenetic protein (BMP) [17]. These factors stimulate the synthesis of the intracellular matrix through different pathways, promote the expression of aggrecan (ACAN) and type II collagen (COL2A1), promote the differentiation of cartilage precursor cells into mature chondrocytes, maintain the chondrocyte phenotype, and inhibit the decomposition of the intracellular matrix [18].

Nerve growth factor (NGF) is a crucial cytokine in the nervous system that obviously influences the growth, development, differentiation, and survival of neurons and has clinical importance due to its ability to regulate nerve regeneration and repair following injury [19]. Adverse effects, such as rapidly progressive osteoarthritis, may occur in some patients treated with anti-NGF antibodies, indicating that the presence of NGF plays an important role in maintaining the process of repairing damaged bone and cartilage [20–23]. NGF has a short half-life in the body and is easily inactivated [24]. Systemic or local administration of a single dose of NGF is not effective and has obvious side effects, for example, pain at the injection site, transient aminotransferase elevation, dizziness, insomnia, and continuous administration is required to maintain its effect. The use of biomaterials for sustained release can maintain the biological activity of NGF in a physiological environment. The double emulsification-solvent volatilization method is the most common and mature method for preparing cytokine-loaded sustained-release microspheres [25, 26].

In this study, NGF-loaded sustained-release microspheres were prepared and incorporated into SF/CS scaffolds carrying BMSCs to obtain NGF-SF/CS-BMSC composites, which were then implanted into cartilage defects in rabbit knees. This study aimed to investigate the effect of NGF-SF/CS-BMSCs on the repair of rabbit knee articular cartilage defects.

## Materials and methods

### Preparation of the SF/CS scaffold

**Fig. 1:**
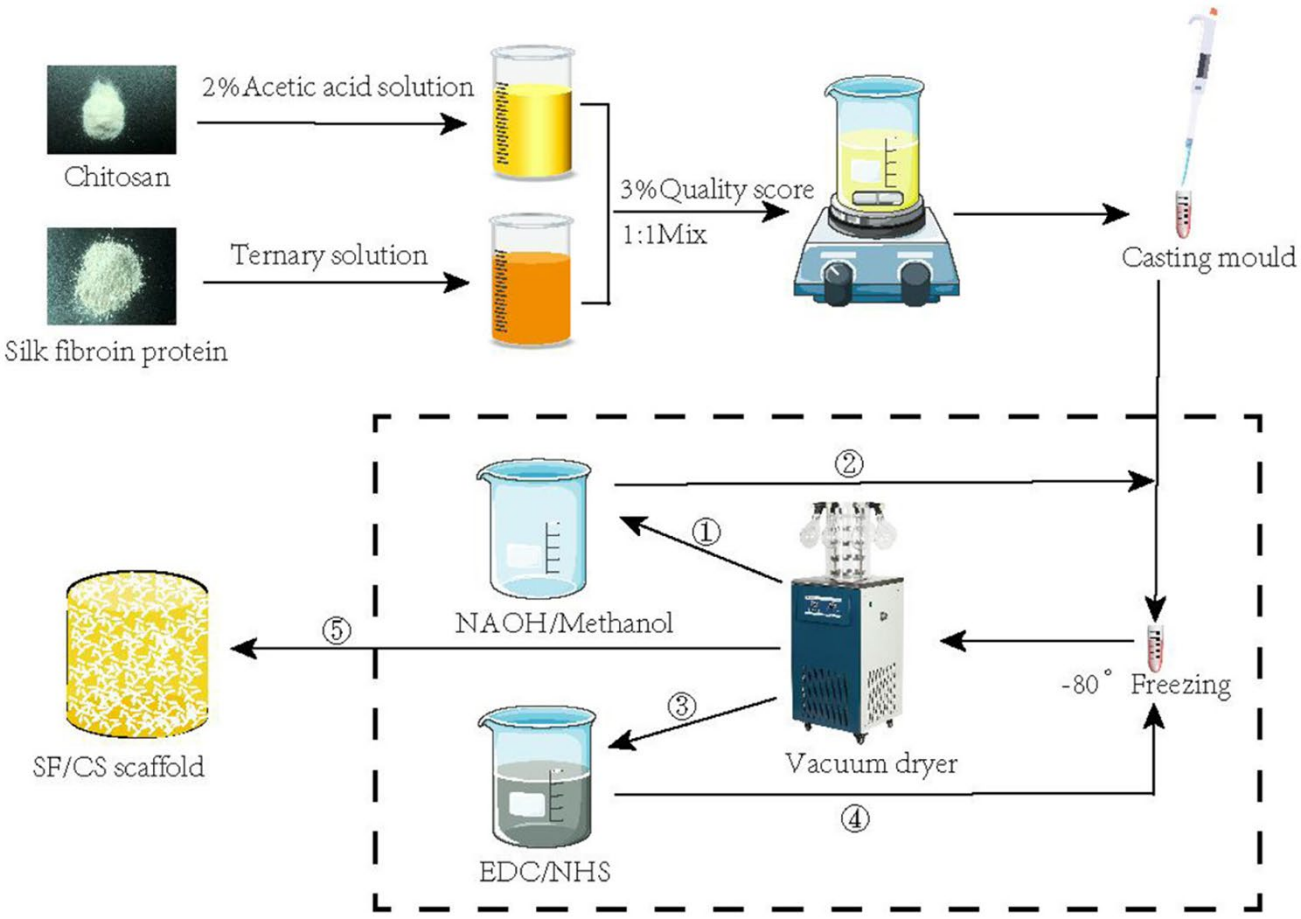
Flow chart of SF/CS scaffold preparation

As shown in Fig. 1, SF powder (Zhejiang, China) was dissolved in a CaCl_2_ (Macklin, Shanghai, China)/H_2_O/C_2_H_5_OH (Macklin, Shanghai, China) ternary solution at a molar ratio of 1:8:2. After extraction and purification, an SF solution at a concentration of 3% was obtained. CS powder (Aladdin Shanghai, China) was dissolved in 2% acetic acid (Kelong, Chengdu, China) to prepare a 3% CS solution. Then, 50 ml of SF solution and 50 ml of CS solution were mixed and stirred continuously with a magnetic stirrer for 1.5 h. The mixture was then poured into a mould and frozen at -80 °C for 24 h, followed by drying in a vacuum freeze dryer (Ningbo Xinzhi Biochemical Co., Ltd., Ningbo, China). The SF/CS scaffolds were immersed in a solution (75% methanol (Kelong, Chengdu, China) and 1 mol/L NaOH (Kelong, Chengdu, China) at a volume ratio of 1:1, 50 mmol/L EDC (Aladdin, Shanghai, China) and 20 mmol/L NHS (Aladdin, Shanghai, China) at a volume ratio of 1:1), crosslinked twice, and then dried under a vacuum. The morphology of the SF/CS scaffold was observed by gross observation, scanning electron microscopy (SEM) and pathological examination.

### Isolation, purification and culture of rabbit BMSCs

**Fig. 2:**
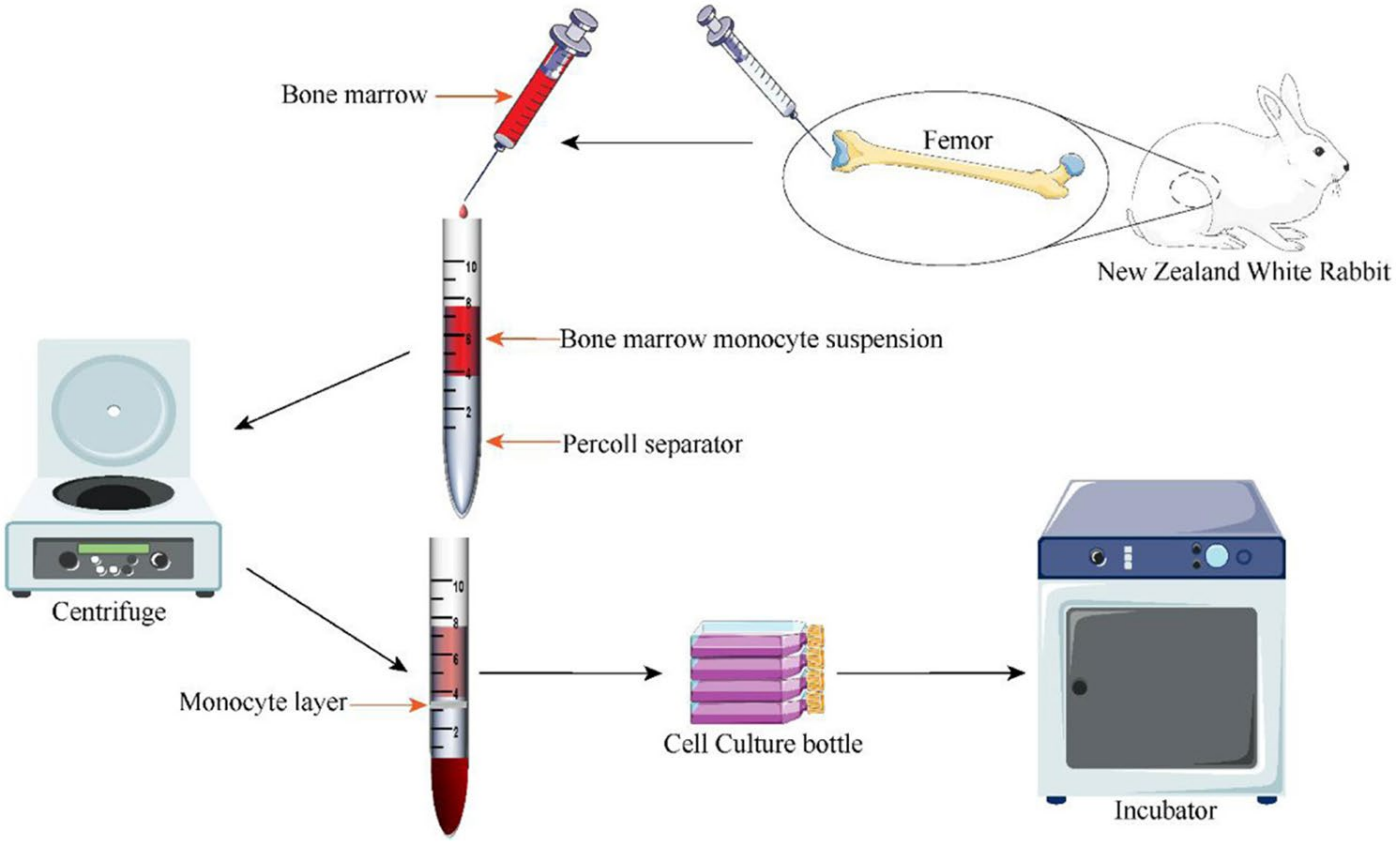
Flow chart of the isolation and culture of BMSCs

All animal procedures were approved by the Institutional Animal Care and Use Committee of The First People’s Hospital of Zunyi (No.2020-1-17). As shown in Figs. 2 and 5- to 6-month-old New Zealand white rabbits (Jinan Jinfeng, China) were anaesthetized. After the operative field was routinely disinfected and covered with a sterile drape, 4 mL of bone marrow was extracted from the bilateral femur and tibia with a syringe containing heparin. Four millilitres of phosphate-buffered saline (PBS) was added, and a cell suspension was prepared to isolate monocyte cells using monocyte isolation solution (Anhui Biosharp, China). Then, the cells were seeded in culture flasks at a density of 1.4 × 10^4^ cells/cm^2^ and placed in a cell culture incubator. The medium was changed every 2–3 days. When the cells were grown to 80–85% confluence, they were digested with trypsin and subcultured at a ratio of 1:2. The subculture methods used were the same as those used for the primary culture. The growth of BMSCs at different time points was observed under a microscope. Third-generation BMSCs were used for subsequent experiments [27, 28].

### Differentiation of BMSCs on the SF/CS scaffold

The sterile SF/CS scaffold was placed in 24-well plates. Well-grown third-generation BMSCs were removed to prepare a cell suspension by adding medium. Then, the cell suspension was seeded onto the scaffold at a density of 5 × 10^5^ cells, and cultured in a CO_2_ incubator with 5% CO_2_ at 37 °C. After 4 h of culture, osteogenic differentiation medium (Procell Wuhan, China) and chondrogenic differentiation medium (ScienCell, USA) were added, and the medium was changed every 2–3 days. After 21 days of culture, the SF/CS scaffold was removed and fixed in 4% paraformaldehyde for 24 h. After dehydration in graded alcohol, embedding in paraffin wax and sectioning, the samples were stained with haematoxylin and eosin (H&E), Alcian blue, and 2% Alizarin red S, after which they were photographed and analysed.

### Preparation and performance evaluation of NGF-loaded sustained-release microspheres

**Fig. 3:**
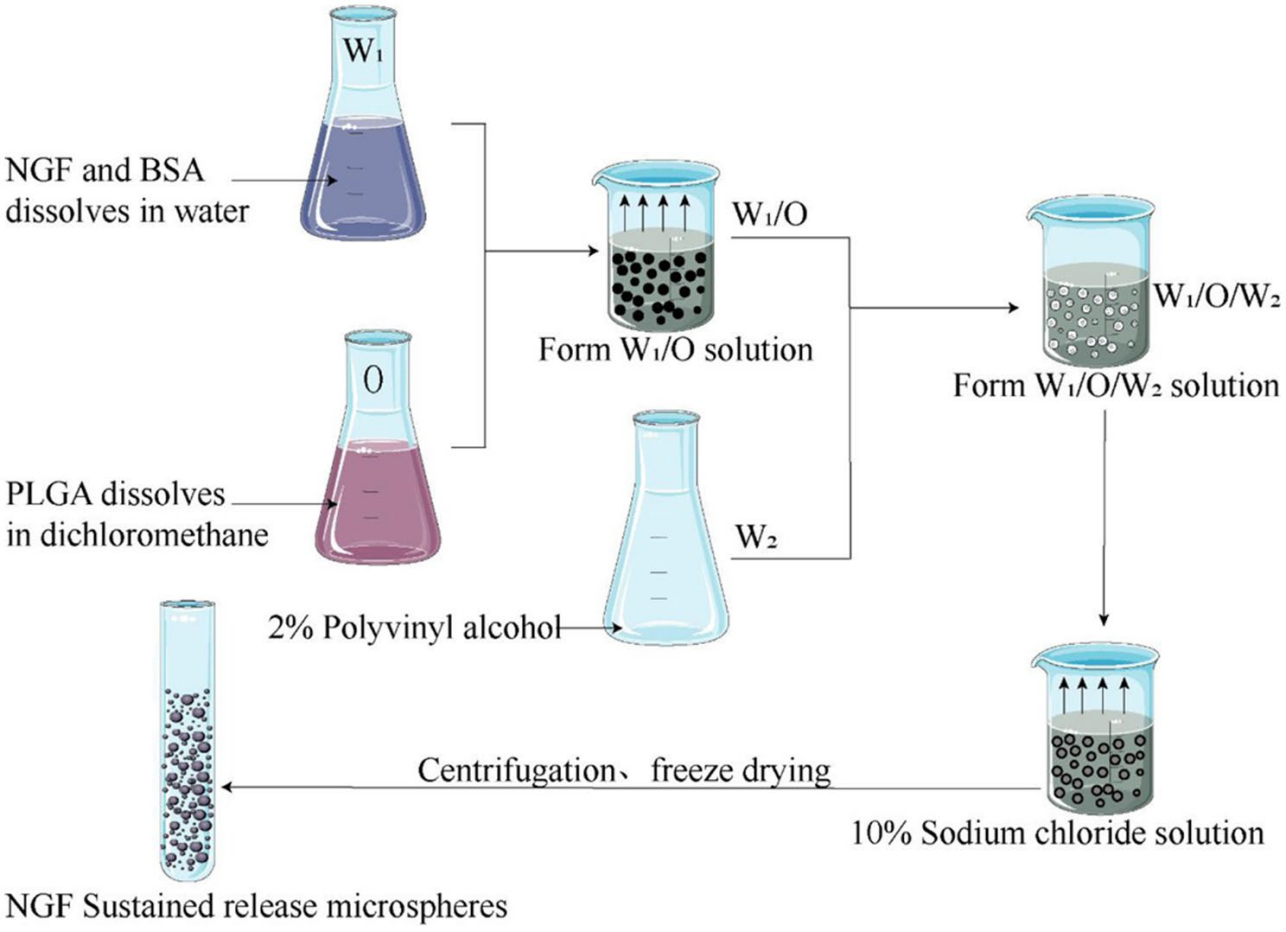
Flow diagram showing the preparation of NGF sustained-release microspheres

As shown in Fig. 3, 100 µg of NGF (Wuhan, China) and 100 mg of bovine serum albumin (Gibco, USA) dissolved in 100 µL of deionized water were used as the internal aqueous phase (W_1_). One hundred milligrams of PLGA (Aladdin, Shanghai, China) dissolved in 2 mL of dichloromethane (Kelong, Chengdu, China) was used as the oil phase (O). The external aqueous phase (W_2_) contained 10 ml of 2% polyvinyl alcohol solution. W_1_ was poured into O, which was then sonicated for 30 s in an ice bath to create the primary emulsion. The primary emulsion was poured into W_2_ and stirred by a magnetic stirrer at 1000 r/min for 30 min in an ice bath to obtain a W_1_/O/W_2_ double emulsion. Then, the double emulsion was poured into 400 ml of 10% deionized water containing sodium chloride solution and stirred by a magnetic stirrer (800 r/min) at room temperature for 4 h to volatilize residual organic solvents. Sustained-release microspheres were collected by centrifugation at 3000 r/min at 4 °C, washed with 100 mL of deionized water 5 times, and freeze-dried in a vacuum freeze dryer for 4 h to obtain NGF-loaded sustained-release microspheres. The above procedure was repeated, and a total of 3 batches of microspheres were prepared. The NGF-loaded sustained-release microspheres were stored at 4 °C (refrigerator) for further experiments. The morphology of the NGF-loaded sustained-release microspheres was observed, and the particle size, encapsulation efficiency, drug loading rate and cumulative release percentage of the microspheres were determined. The NGF-loaded sustained-release microspheres was mixed with PC12 cells to evaluate the biological activity of NGF by counting the number of axons.

### Preparation of NGF-SF/CS-BMSC composites

One hundred milligrams of NGF sustained-release microspheres was dissolved in 1 ml of distilled water and thoroughly mixed. The SF/CS scaffold was placed in a 24-well plate, and 200 µl of the NGF sustained-release microsphere suspension was dropped into the scaffold to ensure that the scaffold was wetted completely. Then, the NGF-SF/CS scaffold composite was frozen in a -80 °C refrigerator for 24 h, dried in a vacuum dryer for 12 h, removed and sterilized with ethylene oxide. Then, the NGF-SF/CS scaffold was placed in a 24-well plate. After the third-generation BMSCs were digested and centrifuged, the cell density was adjusted to 2 × 10^6^ cells/mL. Two hundred microlitres of cell suspension was seeded onto the scaffold and cultured for 4 h. Then, basal medium was added, and the cells were cultured for 24 h. The prepared NGF-SF/CS scaffold-BMSC composite was used in the following experiments.

### Animal experiments

All animal experiments were approved by the Institutional Animal Review Committee of the Third Affiliated Hospital of Zunyi Medical University (No.2020-1-17). All animal experimental procedures were performed under pentobarbital anaesthesia, and efforts were made to minimize animal suffering.

A total of 24 healthy New Zealand white rabbits (male and female, 5–6 months old, weighing 2.5 ± 0.4 kg) were divided into three groups (8 rabbits per group): the NGF-SF/CS-BMSC group (experimental group, treatment of osteochondral defects with the NGF-SF/CS-BMSC composite), the SF/CS-BMSC group (control group, treatment of osteochondral defects with the SF/CS-BMSC scaffold composite), and the SF/CS group (blank group, treatment of osteochondral defects with the SF/CS scaffold). Sample size is determined from the degrees of freedom in ANOVA.

**Fig. 4:**
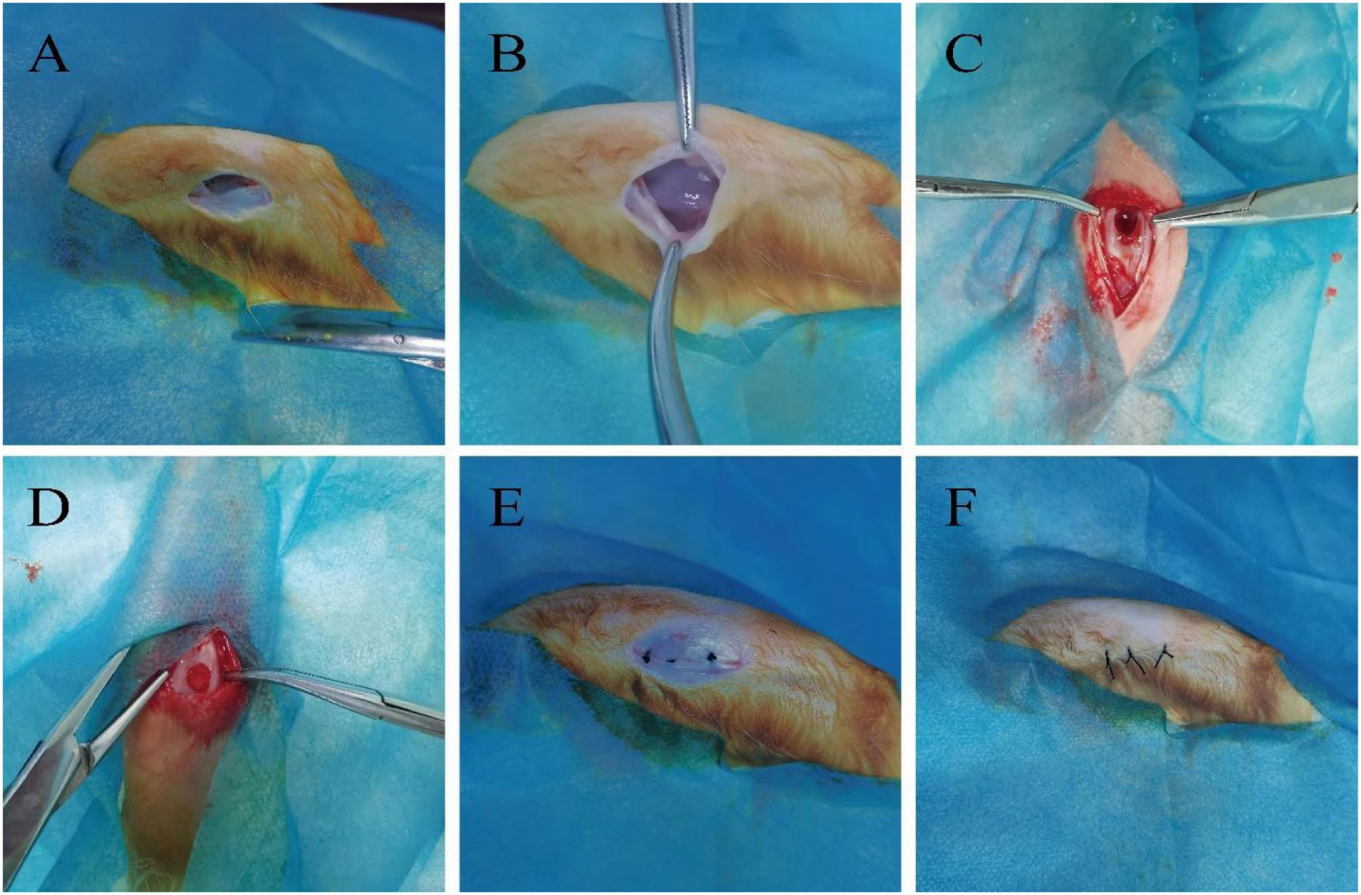
Rabbit cartilage defect model establishment and scaffold implantation. A: 2-cm lateral incision in the knee joint of a rabbit; B: Push the patella inward to expose the intercondylar cartilage of the femur; C: drilling to form osteochondral defects; D: Implant the stent complex in each group; E: Reposition patella, suture lateral collateral ligament of knee joint; F: Intermittent full suture wound

All rabbits were anaesthetized with 2% pentobarbital sodium (1 kg/ml) (Wuhan, China) via the ear vein. After the operation, the field was routinely disinfected and covered with a sterile drape. A 2 cm incision was made over the lateral side of the patella of the bilateral knees. The skin and subcutaneous fascia were incised layer by layer. The patella was dislocated medially. The non-load-bearing region between the femoral condyles was exposed. A osteochondral defect (5 mm diameter, 4 mm depth) [29, 30] was drilled at the non-weight-bearing region of the bilateral femoral condyles using an electric drill to establish a rabbit osteochondral defect model. After washing, the corresponding NGF-SF/CS-BMSCs, SF/CS-BMSCs, and SF/CS were implanted into the defect region of rabbits in each group. After the patella was repositioned, the wound was closed in layers and disinfected with iodophor solution (Fig. 4). After surgery, all rabbits were housed in individual cages, allowed to move freely and had free access to water and food. Gentamicin (4 mg/kg) was injected once daily for 3 days after surgery.

### Observation indicators

At 4, 8, and 12 weeks, computed tomography (CT) (GE, USA), magnetic resonance imaging (MRI) (Siemens, USA) and morphological observation were used to assess the repair of articular cartilage defects, and images were acquired. Then, the knees of the rabbits in each group were harvested. Gross observation of the samples was performed by using the International Cartilage Repair Society (ICRS) scoring system. Pathological stains, such as H&E and Alcian blue, were histologically graded using the modified Wakitani cartilage repair scoring system.

### In vitro experiments

#### The effect of NGF on the proliferation of BMSCs on the SF/CS scaffold

Well-grown third-generation BMSCs were harvested, digested and centrifuged. A cell suspension was made with medium containing 10% serum. Subsequently, the cell concentration was adjusted to 1 × 10^5^ cells/mL, the cells were seeded on the SF/CS scaffold in 96-well plates, and the edge wells were filled with sterile PBS. The control wells contained medium only. The plates were then incubated in an incubator for 4 h, different concentrations of NGF were added, and 5 replicate wells for each concentration were used. Cells in the control wells were cultured with complete culture medium containing 10% serum without NGF. After 24, 48, 72 h and 4, 5, 6, 7, and 8 days of culture, a 96-well plate was removed every day, the medium was changed, and 10 µL of Cell Counting Kit-8 (Keygen, China) solution was added to each well, followed by incubation for 1 h at 37 °C. The medium was then aspirated, and the cells were transferred to another 96-well plate. The absorbance (OD) at 450 nm was determined by a microplate reader. The growth curves of BMSCs cultured with NGF were drawn with the OD value as the ordinate and the time (days) as the abscissa.

### The effect of NGF on the directed chondrogenic differentiation of BMSCs on the SF/CS scaffold

Third-generation BMSCs were harvested, digested and centrifuged. A cell suspension was made with medium containing 10% serum. Then, the cell concentration was adjusted to 5.0 × 10^6^ cells/ml, the cells were seeded on the SF/CS scaffold in 96-well plates, and the edge wells were filled with sterile PBS. The cell suspensions (100 µl) were added to the SF/CS scaffold and cultured for 4 h. Chondrogenic differentiation medium containing different concentrations of NGF was then added, and the medium was changed every 2–3 days. After 7 and 21 days of culture, COL2A1 and ACAN protein expression was determined by western blot analysis, and the mRNA expression of ACAN, SOX9 and COL2a1 was determined by real-time PCR.

### Statistical analysis

Statistical analysis was performed using the SPSS 18.0 software package. All the data are expressed as the mean ± standard deviation (SD). Comparisons between two groups were performed using a *t* test. Comparisons among multiple groups were performed by one-way ANOVA. A value of *P* < 0.05 was considered to indicate statistical significance.

## Results

### Observation of the prepared scaffold

**Fig. 5:**
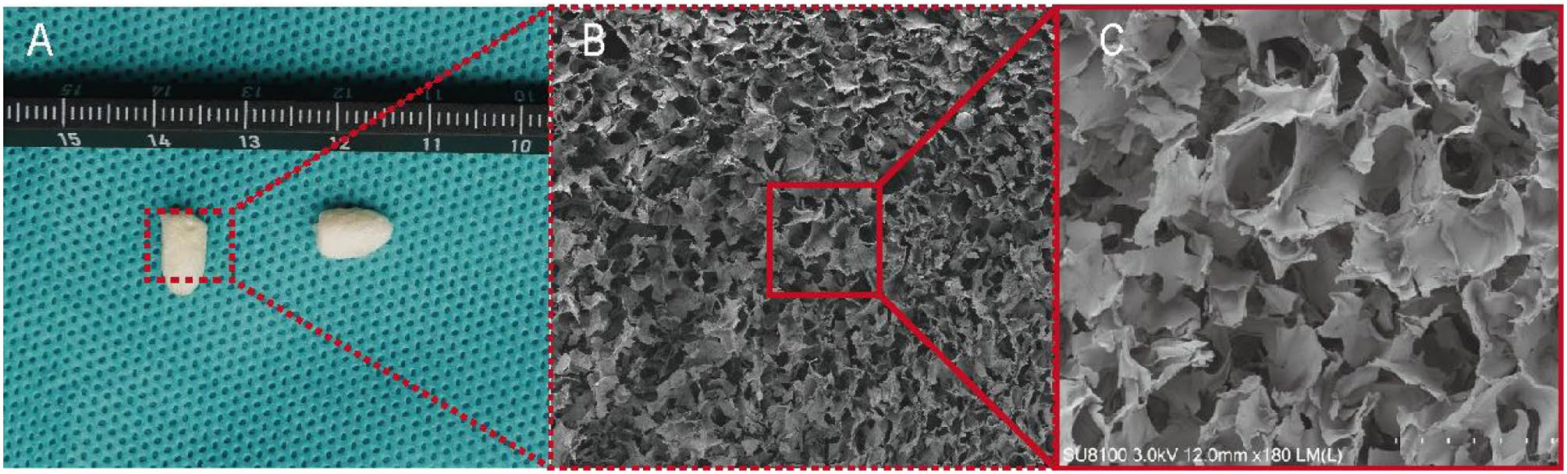
Gross observation and scanning electron microscopy image of the prepared SF/CS scaffold. A: SF/CS support gross observation; B: SEM image of SF/CS scaffold 1 mm ×; C: SEM image of SF/CS scaffold 50 μm ×

**Fig. 6:**
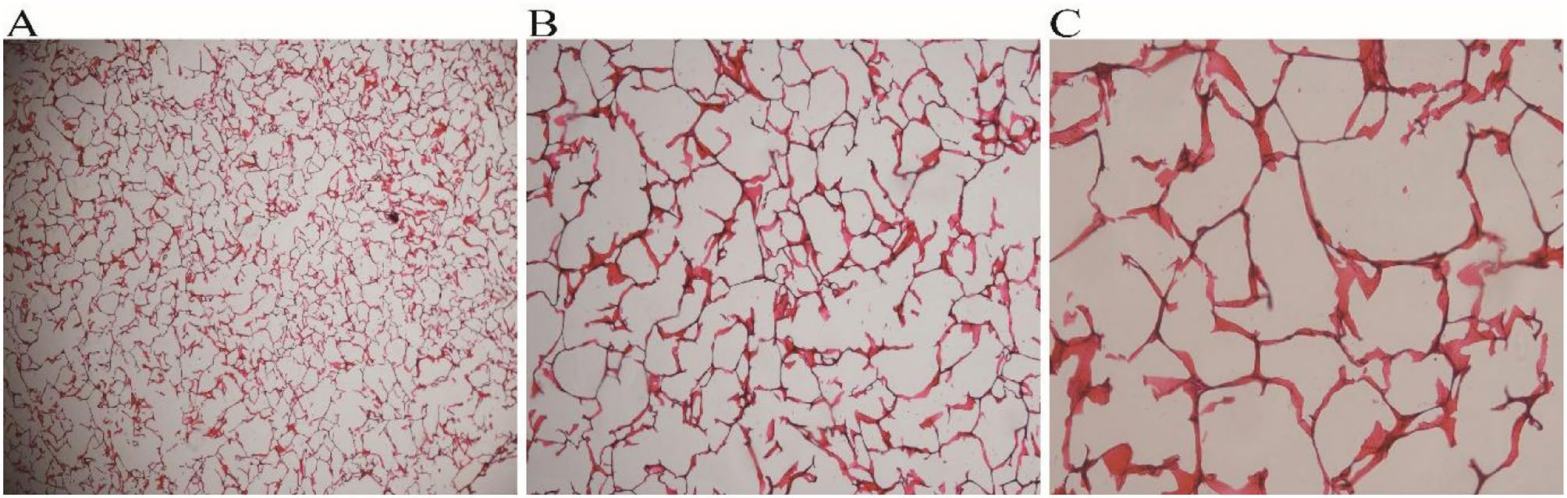
Haematoxylin and eosin staining of the SF/CS scaffold. A: 200 μm×; B: 100 μm×; C: 50 μm×

Gross observation revealed that the SF/CS scaffold was white or milky white, with a regular shape similar to the shape of the mould. The SF/CS scaffold had no special odour, was extremely light, and had obvious pressure resistance and elasticity (Fig. 5A). The transverse SEM images of the SF/CS scaffold showed that the scaffold exhibited a honeycomb shape with irregularly shaped pores (such as polygons and circles), and the pores were highly interconnected (Fig. 5B and C). Histological examination of H&E-stained sections revealed irregular hollow structures inside the scaffold, with pores of varying sizes and thin walls (Fig. 6).

### Morphological observation of rabbit BMSCs

**Fig. 7:**
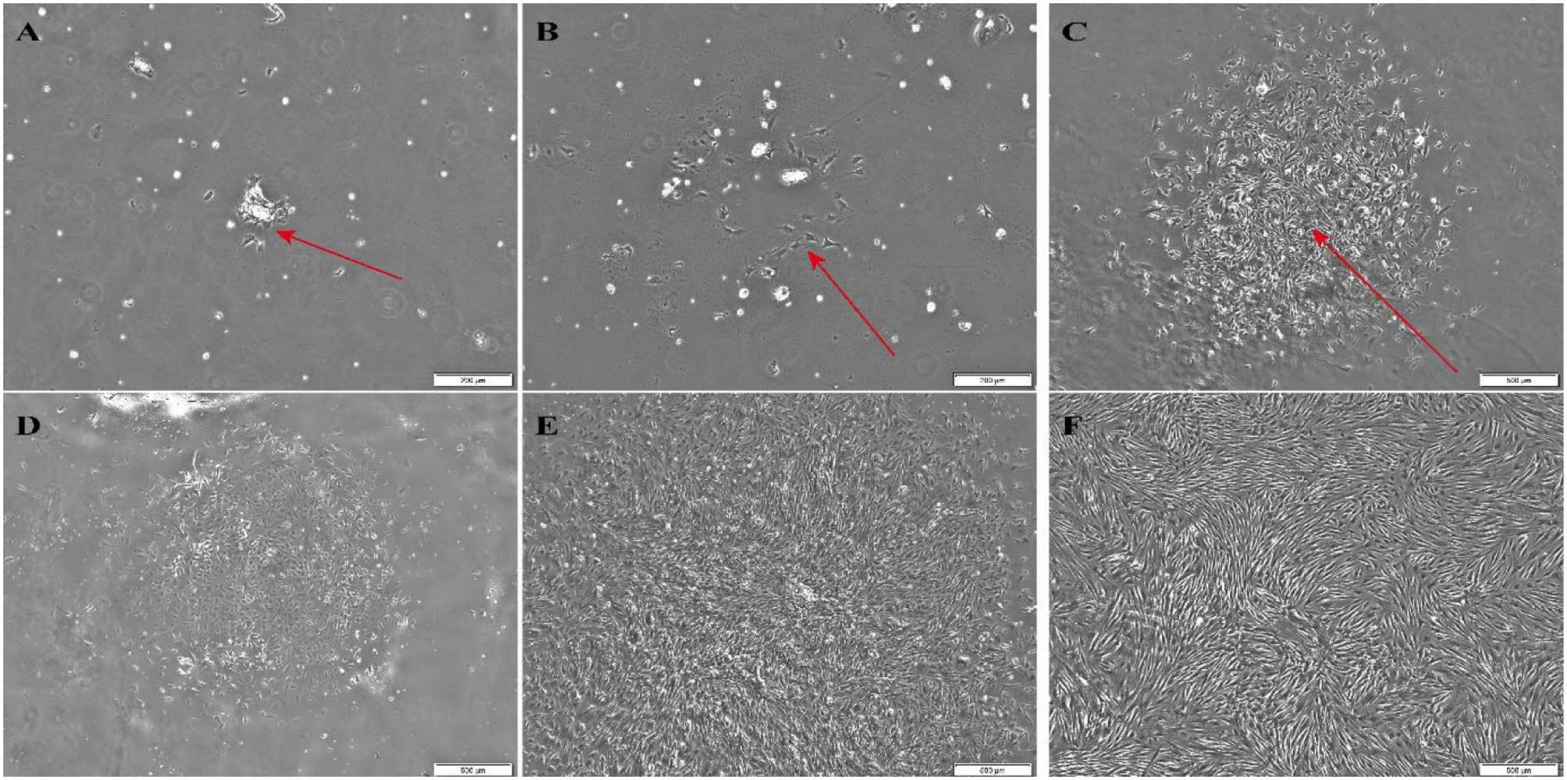
Morphological observation of rabbit BMSCs cultured in vitro after different culture periods. A: 1 day of culture (200×); B: 3 days of culture (200×); C: 7 days of culture (500×); D: morphology of first-generation BMSCs (500×); E: morphology of second-generation BMSCs (500×); F: morphology of third-generation BMSCs (500×)

The adherent BMSCs were predominantly triangular, spindle-shaped or fusiform in shape. After 24 h of culture, a few cells adhered to the bottom of the culture flask, and the cells then gradually became denser and more homogeneous in morphology. After 5 days of culture, the cells gradually grew to confluency and covered the bottom of the flask after 9 days of culture. After generation, second- and third-generation cells were more homogeneous, predominantly spindle or fusiform in shape, and the cells were arranged in parallel and grew in a school-of-fish-like or swirling pattern (Fig. 7).

### Osteogenic and chondrogenic differentiation of BMSCs on the SF/CS scaffold

**Fig. 8:**
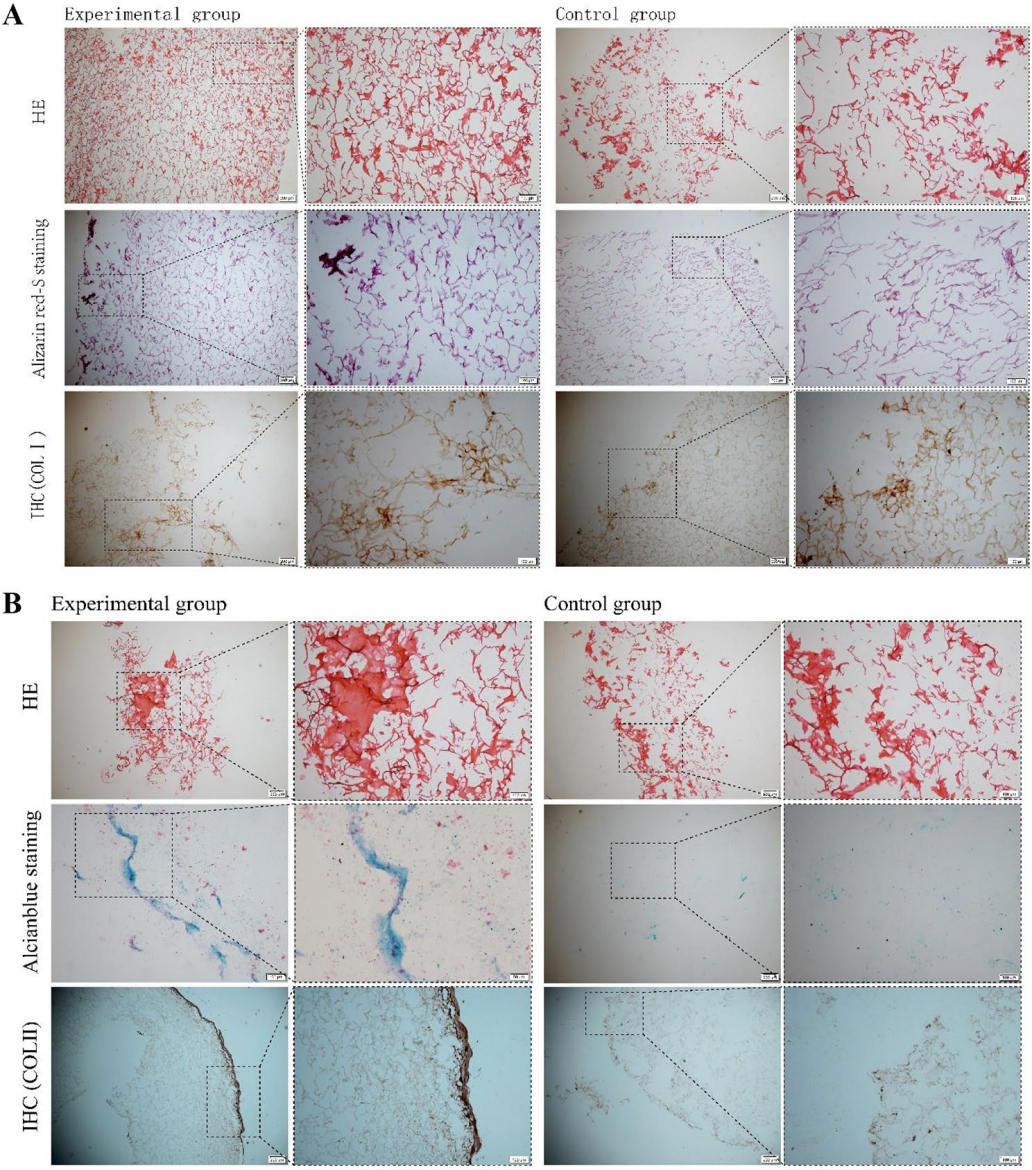
Identification of osteogenic differentiation (A) and chondrogenic differentiation (B) of BMSCs on the SF/CS scaffold

In terms of osteogenic differentiation (Fig. 8A), H&E staining did not reveal differences in the amount of newly formed bone between the experimental and control groups. Alizarin Red staining revealed that the number of calcification nodules in the experimental group was significantly greater than that in the control group. Indicating that rabbit BMSCs showed good compatibility with the SF/CS scaffold and could differentiate into osteocytes and express osteogenesis-related proteins under osteogenic induction.

In terms of chondrogenic differentiation (Fig. 8B), HE staining revealed no significant difference between the experimental and control groups. Alcian blue-positive staining was significantly greater in the experimental group than in the control group. Immunohistochemical staining showed that COL2A1 expression was greater in the experimental group than in the control group. The results also suggested that rabbit BMSCs showed good compatibility with the SF/CS scaffold, could differentiate into chondrocytes, and expressed chondrogenesis-related proteins under chondrogenic induction.

### Morphology and performance of NGF sustained-release microspheres

#### Microsphere morphology observed under light microscopy and SEM

**Fig. 9:**
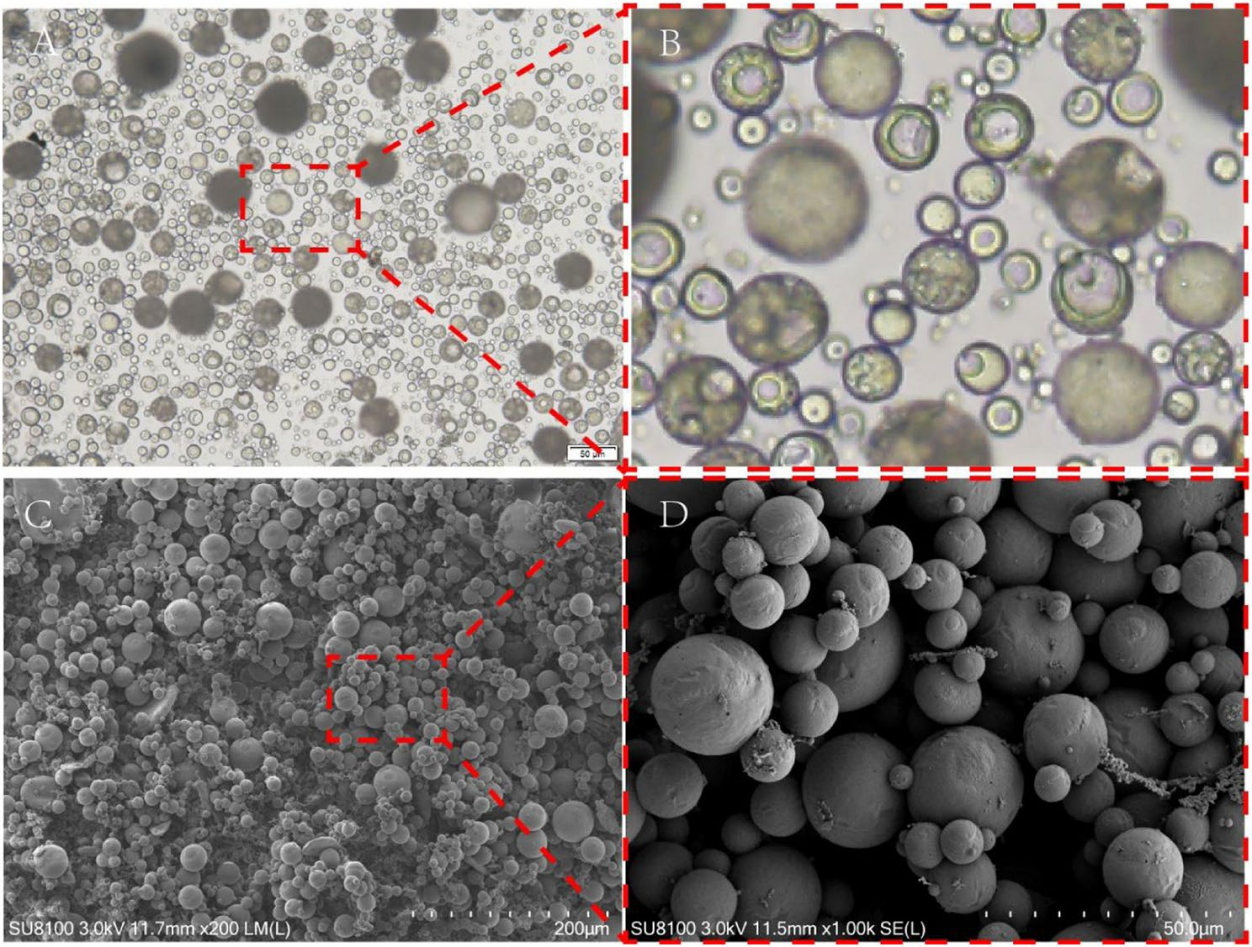
Morphology of NGF sustained-release microspheres observed via light microscopy (A.B) and scanning electron microscopy (C.D)

As shown in Fig. 9, the sustained-release microspheres displayed spherical shapes with different sizes. A high-magnification view of the local area from light microscopy images showed that the microspheres had cores. There was a clear boundary between the core and outer shell. The outer shell of the microsphere was translucent in solution, nearly circular, and intact and provided full encapsulation.

#### The size of NGF sustained-release microspheres

The particle size of the microspheres was determined by a laser particle size analyser (Mastersizer, Malvern, UK), and the size of the sustained-release microspheres varied from 0.461 μm to 756 μm, with a mean particle size of 180 μm. The particle size distribution range was relatively concentrated.

#### Encapsulation efficiency, drug loading rate and cumulative release percentage of the microspheres

**Fig. 10:**
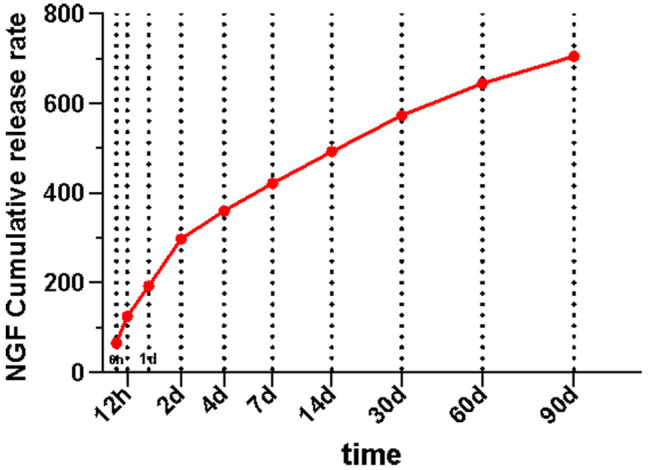
In vitro release profile of sustained NGF release microspheres

As shown in Table 1, the mean mass of the prepared NGF sustained-release microspheres was 99.312 ± 2.725 mg. The encapsulation efficiency and drug loading rate were 31.15% and 31.36%, respectively (Fig. 10).

**Table 1:** Evaluation results of the prepared NGF sustained-release microspheres (mean ± SD, *n* = 3)

List	Value
Total amount of NGF added during preparation (µg)	100
The total mass of sustained release microspheres prepared (mg)	99.312 ± 2.725
The quality of sustained release microspheres was detected (mg)	10
Detected NGF concentration of slow-release microspheres (ng/ml)	603.1 ± 48.138
Detected volume of slow-release microspheres (ml)	5.2
The total mass of NGF in the slow-release microspheres detected (ng)	3136.12

#### Biological activity of NGF

**Fig. 11:**
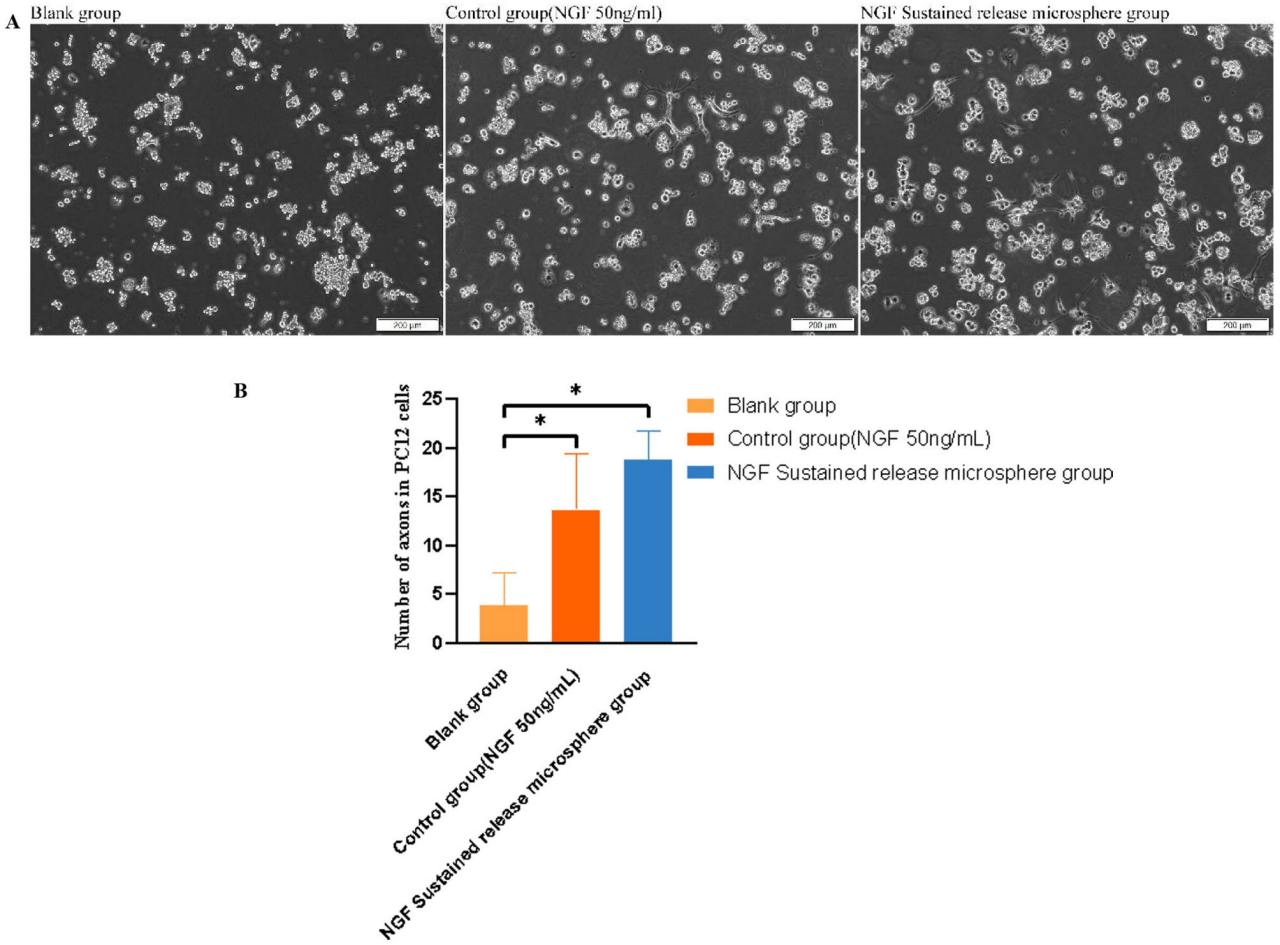
A: Effects of NGF sustained-release microspheres on PC12 cell differentiation; B: Number of axons in PC12 cells in each group

As shown in Fig. 11A, B, after PC12 cells were cultured for 7 days, statistically significant differences in NGF biological activity were found between the blank group and the 50 ng/mL NGF group and between the blank group and the NGF sustained-release microsphere group. There were no statistically significant differences between the 50 ng/mL NGF group and the NGF sustained-release microsphere group, indicating that NGF released by NGF sustained-release microspheres has biological activity.

### Results of animal experiments

#### Imaging findings

**Fig. 12:**
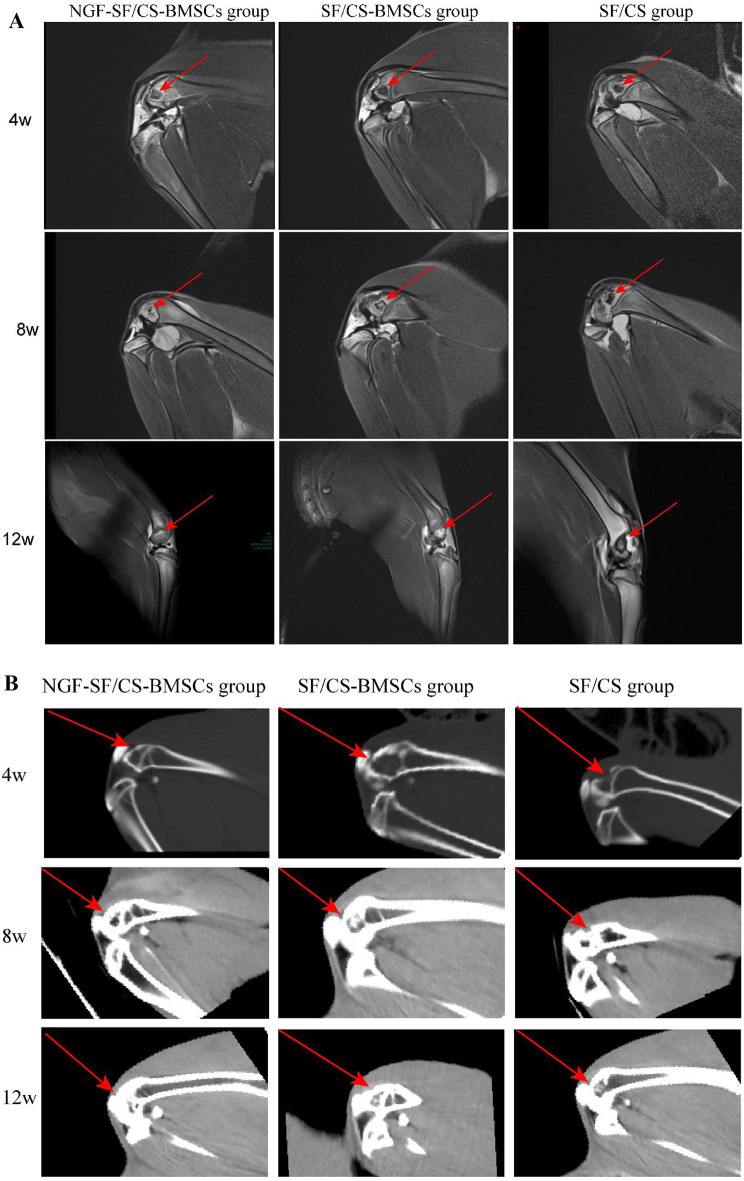
MR images (A) and CT images (B) of knee joints at 4, 8, and 12 weeks after surgery in each group

MRI (Fig. 12A) revealed no obvious cartilage tissue repair in any group at 4 weeks after surgery. At 8 weeks, little newly formed cartilage tissue was observed; the tissue covered the defects of the knee joints in the NGF-SF/CS-BMSC group, but the repair was incomplete. In the SF/CS-BMSC group, little repair tissue was also observed in the cartilage defects, but the amount of local repaired cartilage was less than that in the NGF-SF/CS-BMSC group. In the SF/CS group, the defect was not filled with new bone or cartilage. At 12 weeks, the defect was fully covered by new cartilage tissue in the NGF-SF/CS-BMSC group. Slight depressions were observed in the centre of the cartilage defect in the SF/CS-BMSC group. In the SF/CS group, the defect was not covered by cartilage. CT images (Fig. 12B) showed that at 4 weeks after surgery, a sporadic and scattered distribution of bone tissue was observed in the defects in the NGF-SF/CS-BMSC group. No bone tissue was observed in the defects in either the SF/CS-BMSC or SF/CS group. At 8 weeks after surgery, the newly formed bone tissue completely covered the bottom of the articular cartilage defects in the NGF-SF/CS-BMSC group. Only a small amount of bone tissue was scattered in the defects in the SF/CS-BMSC group. No bone tissue was observed in the defects in the SF/CS group.

At 12 weeks after surgery, cartilage tissue repair in the NGF-SF/CS-BMSC group was similar to that observed at 8 weeks. More bone tissue was observed at the bottom of the knee joint defects in the SF/CS-BMSC group than in the other groups at 8 weeks after surgery. There was almost no bone tissue at the bottom of the defects in the SF/CS group.

#### Gross observation of knee joints of rabbits

**Fig. 13:**
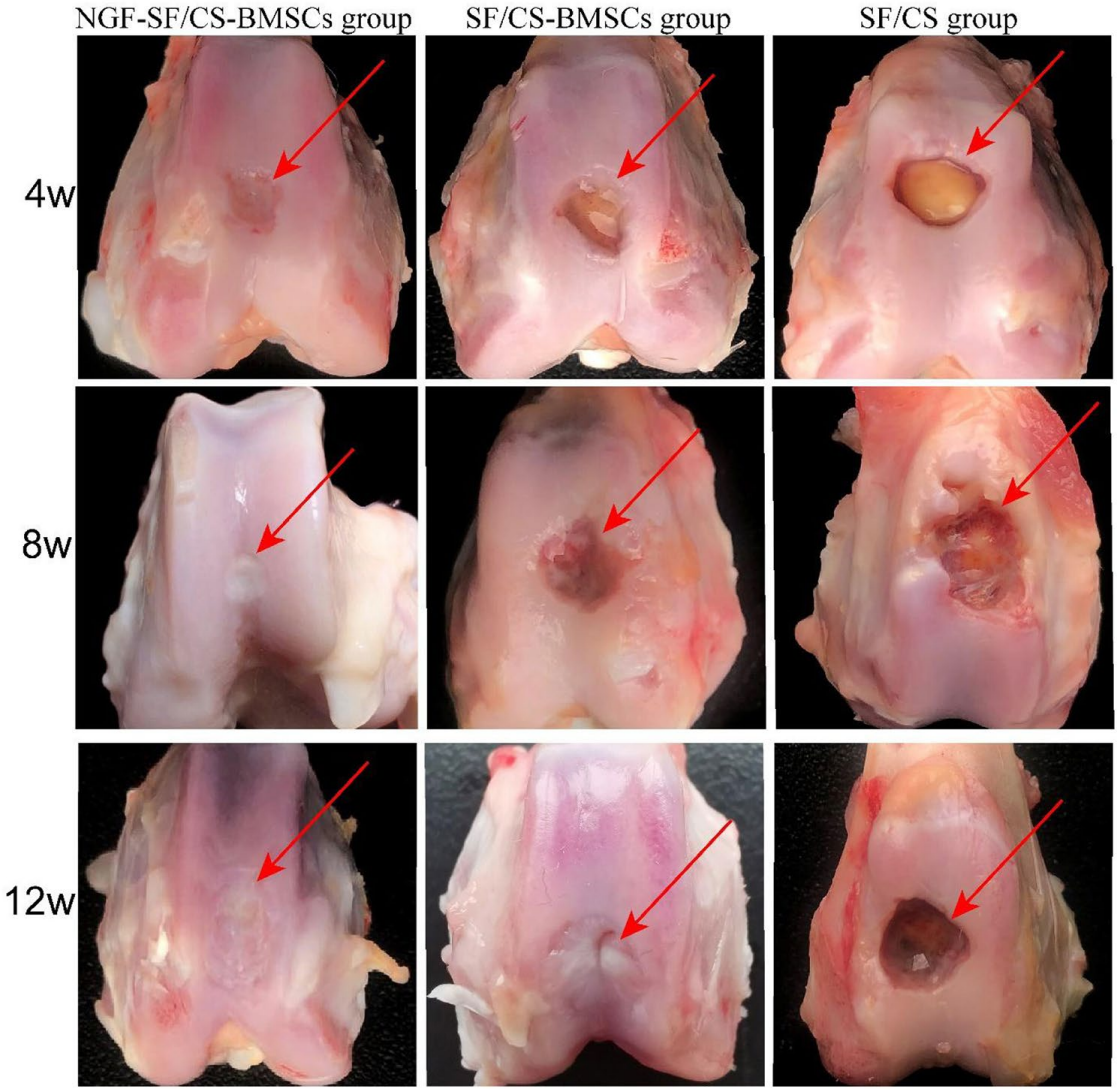
Gross observation of the knee joints of rabbits in each group at 4, 8, and 12 weeks after surgery

**Fig. 14:**
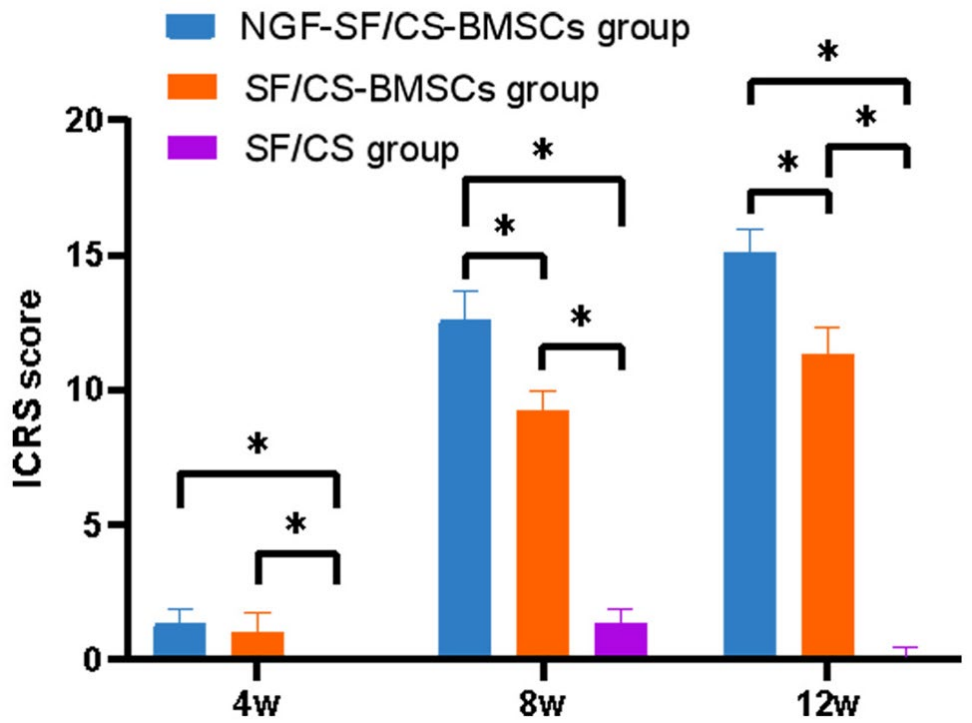
International Cartilage Repair Society (ICRS) score in each group at 4, 8, and 12 weeks after surgery

Gross observation of articular cartilage repair at 4, 8, and 12 weeks after surgery is shown in Fig. 13. At 4 weeks, there was no significant difference in the ICRS between the NGF-SF/CS-BMSC and SF/CS-BMSC groups, whereas significant differences were found between the NGF-SF/CS-BMSC and SF/CS groups. At 8 weeks and 12 weeks, the ICRS scores were significantly greater in the NGF-SF/CS-BMSC group than in the SF/CS-BMSC and SF/CS groups (Fig. 14).

**Fig. 15:**
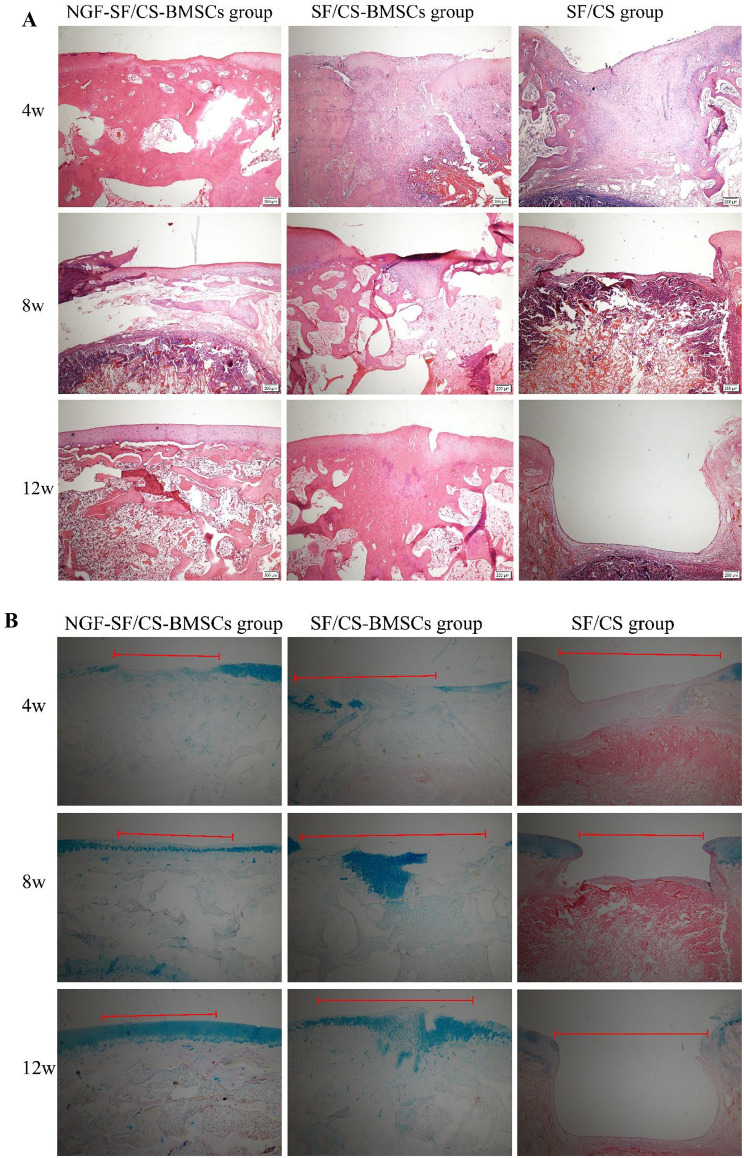
A: Haematoxylin and eosin staining of knee sections from each group at 4, 8, and 12 weeks after surgery. B: Alcian blue staining of knee sections from each group at 4, 8, and 12 weeks after surgery

As shown in Fig. 15A, B, H&E staining and Alcian blue staining revealed no cartilage tissue repair in any of the groups at 4 weeks, but the subchondral bone and underlying trabecular bone tissue were obviously repaired in the NGF-SF/CS-BMSC group. The defects were not repaired in the SF/CS-BMSC or SF/CS group. At 8 weeks, partial cartilage tissue covered the defects in the NGF-SF/CS-BMSC group, and the layers of cartilage and subchondral bone were clear. Cartilage tissue repair was observed in the SF/CS-BMSC group, but the layers of cartilage and subchondral bone tissues were unclear. Cartilage and subchondral bone were not repaired in the SF/CS group. At 12 weeks, the cartilage in the NGF-SF/CS-BMSC group was completely repaired, and the layers of cartilage and subchondral bone were clear. In the SF/CS-BMSC group, the cartilage was partially repaired, and the cartilage tissues were embedded into part of the subchondral bone, with unclear layers. No repair of cartilage or subchondral bone was observed in the SF/CS group.

**Fig. 16:**
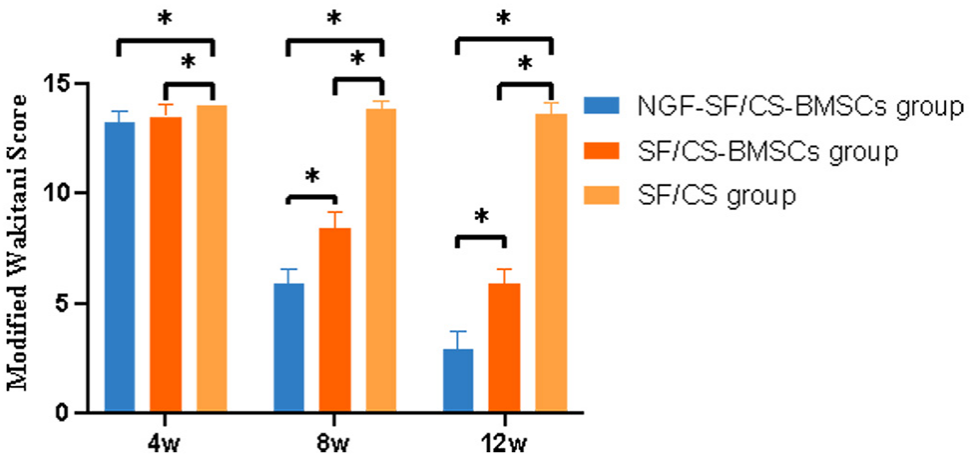
Modified Wakitani scores at 4, 8, and 12 weeks after surgery in each group

Cartilage repair was histologically graded using the modified Wakitani cartilage repair scoring system (Fig. 16). At 4 weeks, there was no statistically significant difference in Wakitani scores between the NGF-SF/CS-BMSC and SF/CS-BMSC groups, while statistically significant differences were found between the NGF-SF/CS-BMSC and SF/CS groups and between the SF/CS-BMSC and SF/CS groups. At 8 and 12 weeks, the Wakitani scores in the NGF-SF/CS-BMSC group were significantly lower than those in the SF/CS-BMSC and SF/CS groups.

### The results of in vitro experiments

#### NGF does not affect the proliferation of BMSCs on the SF/CS scaffold

**Fig. 17:**
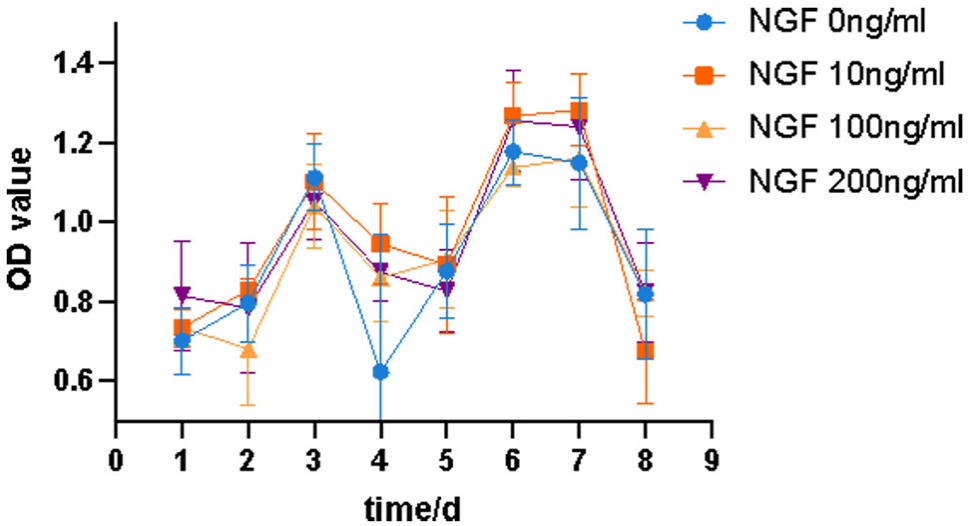
Effects of different concentrations of NGF on the proliferation of BMSCs on the SF/CS scaffold

The proliferation of BMSCs on the SF/CS scaffold fluctuated, but the fluctuation patterns were the same among all groups, and there was no significant correlation between the proliferation of BMSCs and the addition of different concentrations of NGF, indicating that NGF had no effect on the proliferation of BMSCs on the SF/CS scaffold (Fig. 17).

#### NGF exerts no effect on directed chondrogenic differentiation of BMSCs on the SF/CS scaffold

**Fig. 18:**
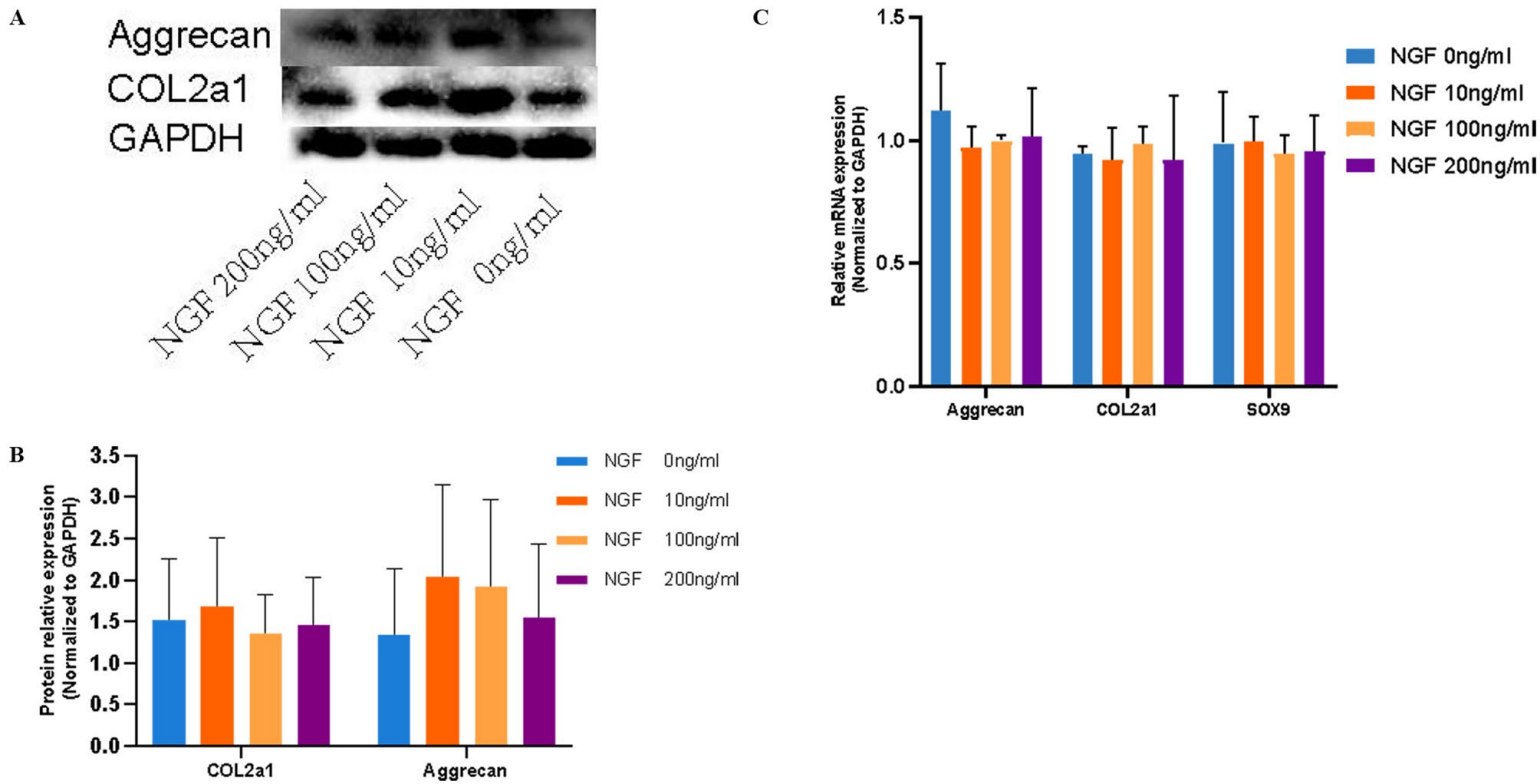
A: Western blot analysis of COL2a1 and ACAN protein expression in chondrocytes differentiated from BMSCs on the SF/CS scaffold after the addition of different concentrations of NGF. B: Graph showing grey values of the COL2a1 and ACAN protein bands. C: mRNA expression of ACAN, COL2a1, and SOX9 detected by RT‒PCR

After 21 days of culture, the expression levels of COL2a1 and ACAN secreted by chondrocytes differentiated from BMSCs on the SF/CS scaffold were detected by western blot analysis, and the results showed that there was no significant difference in the protein expression of COL2a1 and ACAN between the groups after the addition of different concentrations of NGF (Fig. 18A, B).

Similarly, RT‒PCR revealed no significant differences in the mRNA expression of COL2a1, ACAN or SOX9 between the groups. These results indicated that NGF has no obvious promoting or inhibitory effect on the expression of chondrogenic matrix genes (COL2a1 and ACAN) secreted by chondrocytes differentiated from BMSCs on the SF/CS scaffold (Fig. 18C).

## Discussion

Repairing articular cartilage after injury is challenging, and tissue engineering is a potential solution [10]. Previous experiments have demonstrated that SF/CS scaffolds, when combined with bone marrow mesenchymal stem cells, exhibit a beneficial effect on the repair of rabbit articular bone cartilage defects. In order to enhance this effect, cytokine NGF was added, and our experimental grouping was determined by this rationale. In this study, a composite of slow-release microspheres containing nerve growth factor (NGF), silk fibroin/chitosan (SF/CS) scaffolds, and bone marrow-derived mesenchymal stem cells (BMSCs) was implanted into the knee joints of rabbits with endochondral defects to evaluate its effects. Imaging and pathology revealed that at 8 and 12 weeks, the experimental group exhibited significantly better cartilage repair than the control and blank groups. The repaired cartilage tissue in the experimental group was similar in structure and thickness to the original articular cartilage. Our findings suggest the SF/CS scaffolds with NGF slow-release microspheres and BMSCs had better reparative effects on rabbit joint defects, providing a promising approach for articular cartilage repair.

In vitro experiments revealed that NGF did not significantly promote or inhibit the proliferation of BMSCs, and NGF did not promote the expression of cartilage matrix by BMSCs or differentiated chondrocytes. As shown by our in vivo results, especially at 4 w, no obvious cartilage tissue repair was observed in any of the groups, but in the experimental group, there was obvious subchondral bone tissue generation in the cartilage. At 8 and 12 w, articular cartilage repair gradually occurred, whereas in the control group, although there was repair of subchondral bone at 8 w, incomplete articular cartilage repair was observed at 12 w. Therefore, we speculate that NGF-SF/CS-BMSCs promote the repair of rabbit knee joints, probably not by promoting the proliferation of BMSCs and directional chondrogenic differentiation following the expression of COL2a1 and ACAN but rather by promoting the repair of subchondral bone at an early stage, which in turn promotes the repair of articular cartilage.

Many experimental studies have confirmed that NGF plays a role in promoting osteogenesis and can promote bone tissue regeneration, chondrocyte hypertrophy and differentiation, and the formation of osteoblasts, but few studies have investigated the role of NGF in promoting cartilage repair. At present, it is considered that the use of bionic composite scaffold with mesenchymal stem cells can promote chondrocyte differentiation; the direct promotion of the transformation of BMSCs into chondrocytes; the maintenance of the chondrocyte phenotype; the delay of chondrocyte hypertrophic differentiation; or the promotion of COL2a1 and ACAN expression to achieve cartilage tissue repair. Results from our experiments, including both in vivo and in vitro experiments showed that NGF did not promote the transformation of BMSCs into chondrocytes and had no promotional effect on the chondrocyte expression of COL2a1 or ACAN. NGF may not promote articular cartilage repair by promoting subchondral bone repair.

It has long been believed that degeneration and wear and tear of articular cartilage are the main causes of cartilage damage and defects, and many studies on the repair and treatment of cartilage defects have focused on the cartilage layer, often ignoring the importance of the subchondral bone [31, 32]; clinical studies have shown that the subchondral bone of patients with osteoarthritis is altered earlier than the articular cartilage [33, 34], which suggests that the integrity of the subchondral bone and the process of remodelling are important.

In particular, the use of the new drug tanezumab (NGF antibody) has been found to cause rapidly progressive osteoarthritis, which requires immediate joint replacement treatment in severe cases. The specific reason for this is still unclear, and in combination with the experiments, we speculate that it may be related to the fact that tanezumab mainly interferes with the homeostasis of the subchondral bone, which in turn affects the stability of the subchondral bone, leading to the rapid progression of osteoarthritis. Therefore, future studies need to focus more on the reconstruction and repair of the subchondral bone [35].

The grouping design of this experimental study was based on the preliminary analysis of the repair effect of NGF on articular cartilage with the SF/CS scaffold composite BMSCs, so the NGF slow-release microsphere group and the blank control group were not separately established. Second, the number of slow-release microspheres in vivo was not further tested, which was mainly considered to indicate that the slow-release microspheres are affected by various factors in vivo. Third, this study investigated the reparative effect of NGF on articular cartilage, but NGF is an important biological factor for pain generation [36], and no pain-related indices were measured.

## Conclusions

In summary, this study clarified that SF/CS scaffolds containing NGF slow-release microspheres combined with BMSCs have better effects on the repair of articular osteochondral defects, and this reparative effect may be related to the promotion of subchondral bone repair at an early stage.

## Data Availability

All the data generated or analysed during this study are included in this published article.

## Abbreviations

SF: Silk Fibroin
CS: Chitosan
BMSCs: Bone Marrow Mesenchymal Stem Cells
NGF: Nerve Growth Factor
SEM: Scanning Electron Microscopy
PBS: Phosphate-Buffered Saline
CT: Computed Tomography
MRI: Magnetic Resonance Imaging
ICRS: International Cartilage Repair Society
OD: Optical Density
ACAN: Aggrecan
